# Visual Navigation during Colony Emigration by the Ant *Temnothorax rugatulus*

**DOI:** 10.1371/journal.pone.0064367

**Published:** 2013-05-09

**Authors:** Sean R. Bowens, Daniel P. Glatt, Stephen C. Pratt

**Affiliations:** 1 School of Life Sciences, Arizona State University, Tempe, Arizona, United States of America; 2 Department of Ecology and Evolutionary Biology, Princeton University, Princeton, New Jersey, United States of America; Stanford University, United States of America

## Abstract

Many ants rely on both visual cues and self-generated chemical signals for navigation, but their relative importance varies across species and context. We evaluated the roles of both modalities during colony emigration by *Temnothorax rugatulus*. Colonies were induced to move from an old nest in the center of an arena to a new nest at the arena edge. In the midst of the emigration the arena floor was rotated 60°around the old nest entrance, thus displacing any substrate-bound odor cues while leaving visual cues unchanged. This manipulation had no effect on orientation, suggesting little influence of substrate cues on navigation. When this rotation was accompanied by the blocking of most visual cues, the ants became highly disoriented, suggesting that they did not fall back on substrate cues even when deprived of visual information. Finally, when the substrate was left in place but the visual surround was rotated, the ants' subsequent headings were strongly rotated in the same direction, showing a clear role for visual navigation. Combined with earlier studies, these results suggest that chemical signals deposited by *Temnothorax* ants serve more for marking of familiar territory than for orientation. The ants instead navigate visually, showing the importance of this modality even for species with small eyes and coarse visual acuity.

## Introduction

Ants rely heavily on vision for navigation, both for detecting celestial cues that guide path integration and for learning and recognizing the details of their surroundings [1]–[5]. At the same time, many ant species deposit and respond to chemical signals that guide them to food sources, nest sites, battlegrounds, and other areas of interest [6]. The interaction of these distinct sources of information has been studied for only a few species. Many experiments show that visual cues predominate over odor marks when the two are placed in conflict, with the latter serving to guide naïve ants that have not yet learned visual cues, or as a backup if visual information is absent [7]–[12]. The two modalities may also act synergistically, allowing more accurate and speedy navigation than visual cues or scent marks alone [13]–[15].

Most previous work has focused on ants that form well-defined odor trails, but chemical cues can be important even for those that do not, such as species in the genus *Temnothorax* [12], [16]–[19]. In experiments with Y-shaped bridges, *T. unifasciatus* foragers prioritized visual cues when these were put in competition with substrate markings, but they resorted to the latter when familiar visual cues were removed [16]. Both kinds of cue were also found to matter during colony emigration, when a corps of transporters must navigate repeatedly between the old and new nests [20]–[22]. Transporters in *T. albipennis* colonies aimed toward a conspicuous visual beacon [18] and also made use of a prominent wall that paralleled the route, apparently choosing their path to keep the wall's image at a memorized retinal position [17]. Evidence for substrate cues is less direct. When an acetate sheet carpeting the floor of an experimental arena was removed midway through an emigration, transporters encountering the uncovered area were reluctant to enter it [17], [18]. A similar effect was seen for *T. affinis* transporters required to cross a narrow bridge: when a familiar bridge was replaced by an unfamiliar one, they refused to cross it [19]. These observations imply that ants leave chemical marks as they explore or follow the route, but the origin and nature of these marks are not known. A more recent experiment suggests that they are less important than visual cues for navigation: colonies were able to find a familiar nest more rapidly than an unfamiliar one, even when substrate cues were exchanged between the routes to the competing nests [12].

The relationship between visual and chemical cues in *Temnothorax* orientation remains uncertain. If visual cues predominate, what role do the chemical marks play? Are they a backup used when visual cues are missing or ambiguous? Or do they simply mark familiar territory, playing little direct role in orientation, as is the case for many ants [23]–[26]? These questions can best be answered by placing visual and chemical cues in conflict and observing which cue guides the subsequent orientation of ants. Past uses of this approach were inconclusive, either because the specific paths taken by navigating ants were not traced [12], or because ants were tested in simplified environments [16], [19]. A Y-bridge, for example, imposes an artificial dichotomy to orientation choices that are likely to be much less constrained in nature, where *Temnothorax* are not known to form branching trail networks. If chemical marks are instead deposited more evenly over the substrate, their role may be very different from that suggested in a forced choice between narrow pathways.

In this study, we examined the relative roles of visual and substrate-borne cues in a more natural geometry, where ants chose their own path across a planar surface. We studied navigation by emigrating colonies of *T. curvispinosus* [20], [27]–[29] in laboratory arenas where we could independently manipulate visual and substrate-bound cues.

## Experimental Designs and Results

### Experiment 1: Substrate rotation does not affect orientation

**Figure 1:**
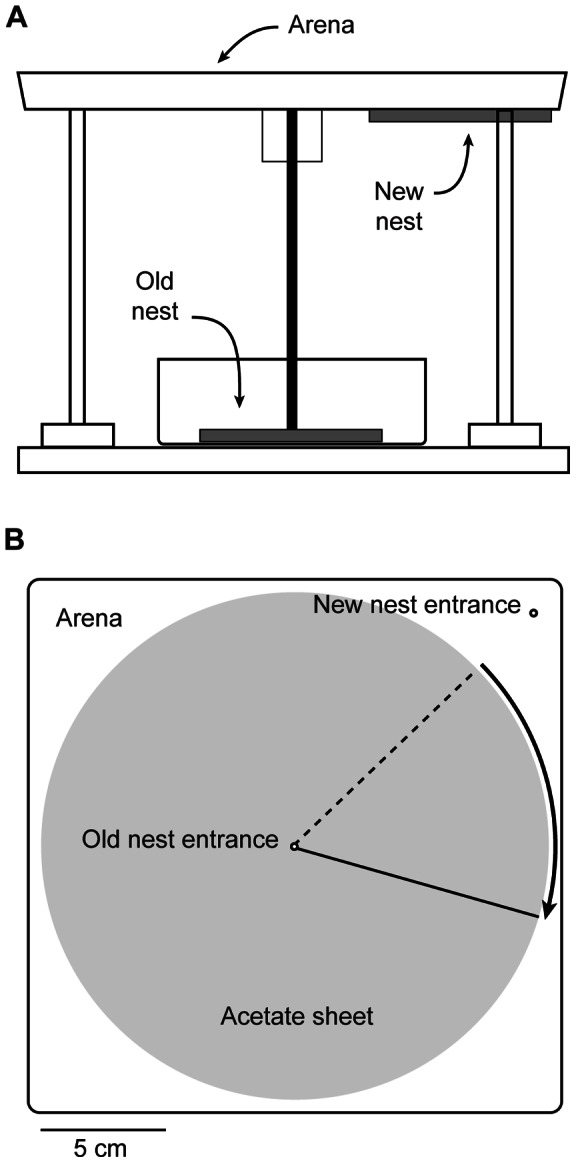
Apparatus for manipulation of substrate cues during colony emigration. A) Side view shows a box containing the old nest below an arena mounted on risers. The new nest was fastened to the bottom of one corner of the arena, which ants reached by climbing a wooden dowel. A Fluon-coated collar guided the ants through an entrance hole to the upper surface of the arena. B) Top view shows the arena floor carpeted with a transparent acetate sheet. In the midst of each emigration, the sheet was rotated 60° clockwise. Emigrating ants were observed to determine if they continued to walk toward the new nest (dashed line) or shifted their headings to match the new substrate position (solid line).

To test the effect of substrate-bound cues, we induced colonies of individually marked ants to emigrate from the center of an arena to a new nest at the arena's edge (Figure 1). We noted the identity of each ant that recruited a nestmate from the old nest to the new. Once an ant had completed three recruitment trips we waited until she returned to the old nest to retrieve another recruit. While she was there, an acetate sheet carpeting the arena floor was rotated 60° clockwise. If ants were guided by substrate-bound cues, we predicted that the focal ant, when she re-emerged from the old nest, would take a path that deviated 60° clockwise from the true bearing of the new nest. If substrate-bound cues were not important, her heading should be unchanged from before rotation.

**Figure 2:**
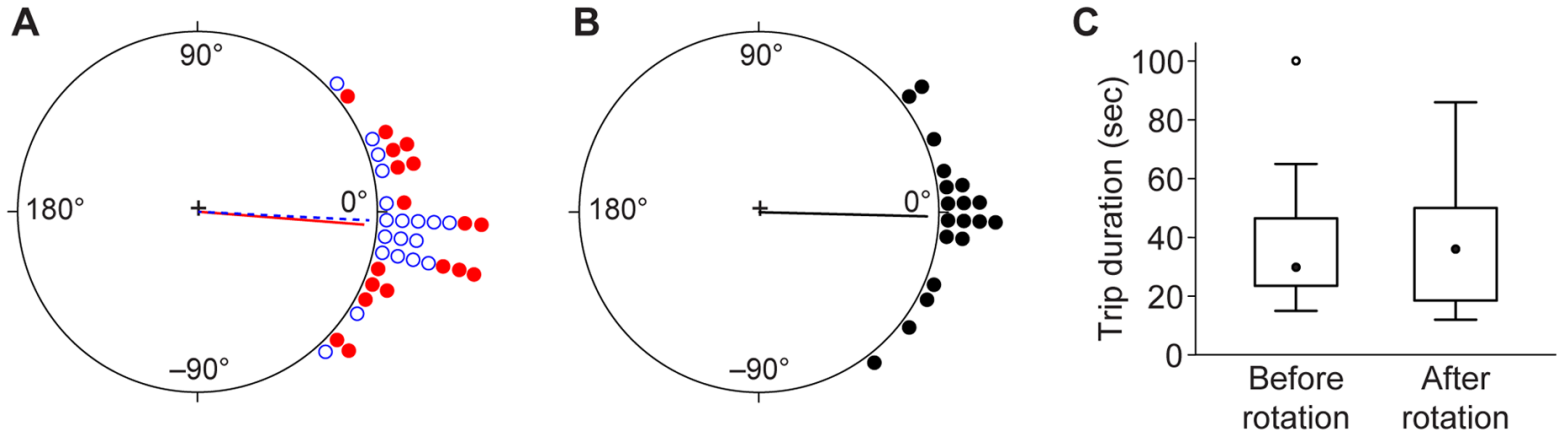
Substrate rotation does not affect orientation by emigrating ants. A) The last trips before rotation (dashed blue line and open blue circles) were highly directed (Rayleigh test: r = 0.95, p = 6×10^−8^), with an average heading only 3° from the direction of the new nest (0°). After rotation (solid red line and closed red circles), headings remained highly directed (Rayleigh test: r = 0.93, p = 9×10^−8^), with an average value only 4° from the nest direction. The difference in mean headings before and after rotation was not significant (Watson's two-sample test: U^2^  = 0.1541, 0.10> p>0.05, n = 19). B) The mean change in heading by each ant could not be distinguished from zero (Hotelling test: F_2, 17_  = 0.32, p = 0.73). C) The duration of journeys from the old to the new nest was not affected by rotation (Wilcoxon paired ranks test: V = 62.5, n = 19, p = 0.30). In each box plot the closed circle shows the median, boxes delimit the 1^st^ and 3^rd^ quartiles, and whiskers show the range. The open circle is an outlier.

**Figure 3:**
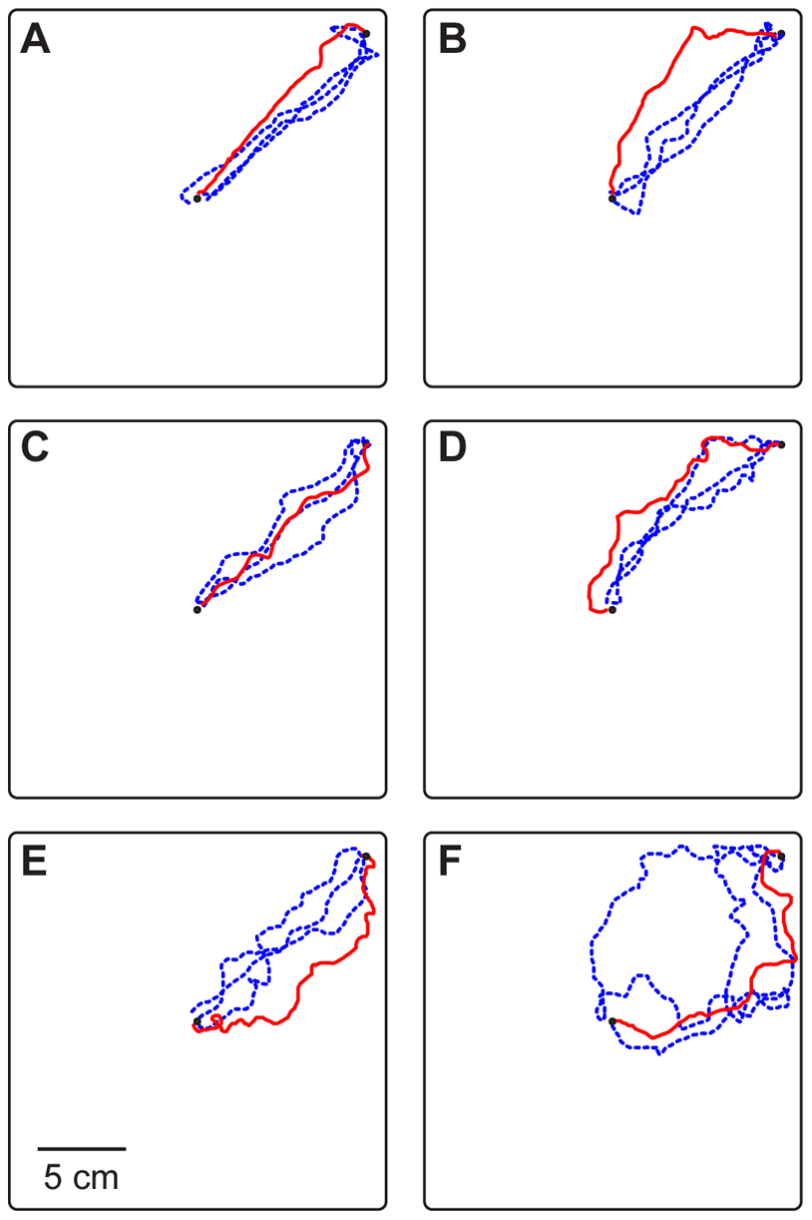
Sample paths before and after substrate rotation. Each panel shows a single ant's paths before rotation (dashed blue line) and afterwards (solid red line). Squares show the arena boundary. Small closed circles show locations of old nest entrance at arena center and new nest entrance at upper right corner.

The results showed no effect of substrate rotation. Before rotation, ants took fairly direct paths, with an average heading that deviated less than 3° from the true bearing of the new nest. After rotation, average heading remained directed toward the nest, rather than shifting to match the new position of the substrate (Figure 2A). Individual headings varied over a range of almost 90°, which might have concealed a true effect of rotation. However, this possibility was not supported by analysis of the change in heading by each ant, which averaged only 1.4° (Figure 2B). There was also no difference in trip duration before (36±21 sec) and after (39±21 sec) rotation (Figure 2C). Finally, inspection of individual paths showed no obvious change in straightness or directedness after substrate rotation (Figure 3).

### Experiment 2: Abolishing visual cues causes disorientation

We hypothesized that the absence of a substrate rotation effect might show a prioritization of visual cues over substrate-borne ones. That is, the ants may attend to substrate cues only if visual cues are not available. To test this, we repeated our first experiment with one change: at the same time that the substrate was rotated, the arena was surrounded with a cylinder of white poster paper that concealed most visual cues that had been available to the ants.

**Figure 4:**
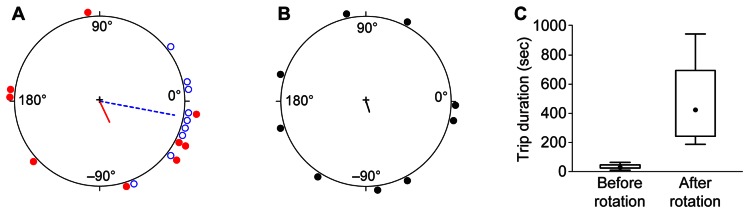
Substrate rotation plus blocking of visual cues causes disorientation. A) Before rotation (dashed blue line and open blue circles), headings were significantly directed (r = 0.89, p = 8×10^−5^) and average heading deviated 11° from the direction of the new nest (0°). After rotation (solid red line and closed red circles), headings were highly dispersed, and the mean heading could not be distinguished from random (average heading  = −65°, r = 0.27, p = 0.53). B) The change in heading was extremely variable across ants and the mean angular change could not be distinguished from random (average change  = −72°, r = 0.13, p = 0.86). C) The duration of journeys from the old to the new nest increased sharply after blocking of visual cues (Wilcoxon paired ranks test: V = 0, n = 8, p = 0.0078). See Figure 2 for box plot details.

**Figure 5:**
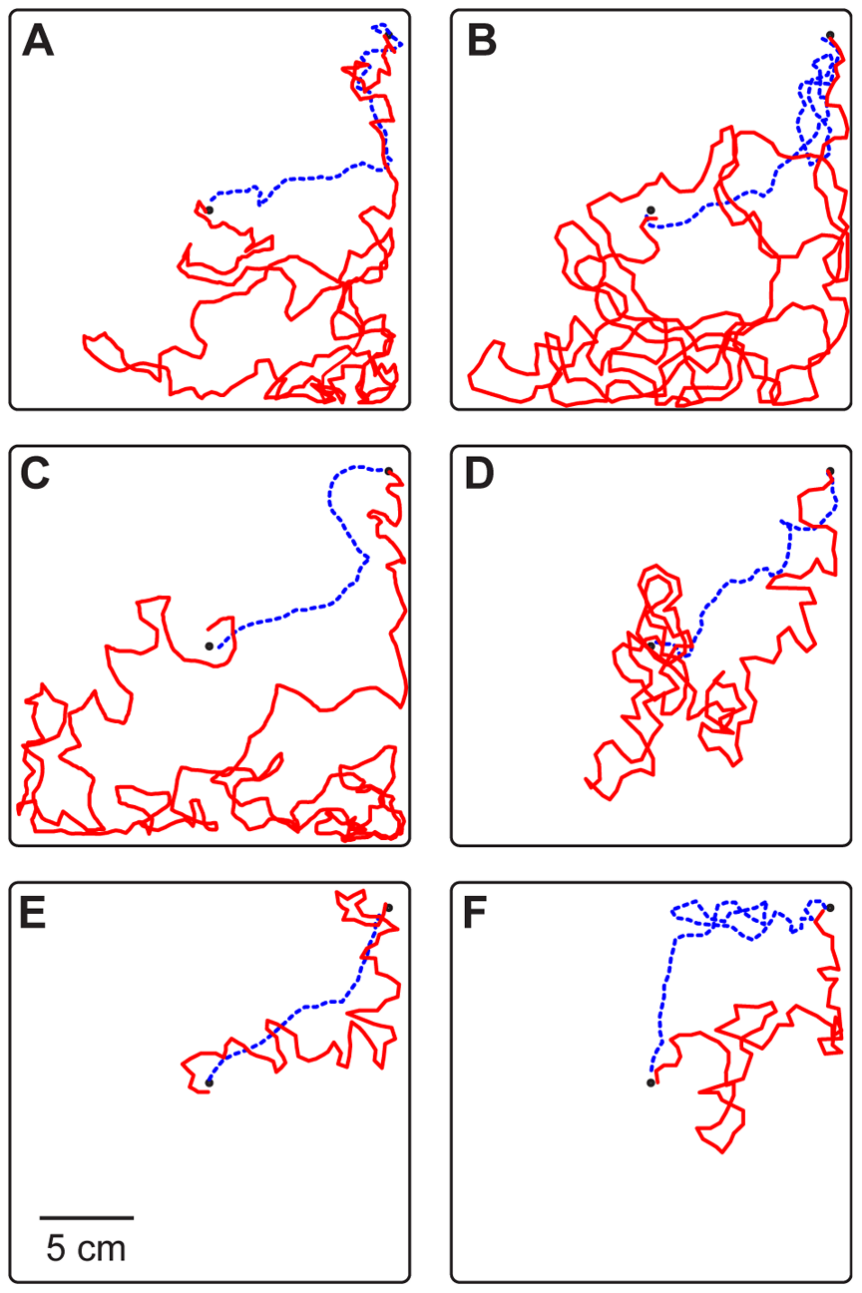
Sample paths before and after substrate rotation plus blocking of visual cues. Each panel shows a single ant's paths before manipulation (dashed blue line) and afterwards (solid red line). Squares show arena boundary. Small closed circles show locations of old nest entrance at arena center and new nest entrance at upper right corner.

The results did not support a role for substrate cues, even after the loss of visual information. Rather than shift their headings to match the new substrate position, the ants showed a dramatic loss of orientation. Average heading changed from strong directedness toward the new nest to random dispersion in all directions (Figure 4A). The change in heading by each ant was highly variable and could not be distinguished from a random distribution (Figure 4B). Trip durations increased from 34±17 sec to 480±300 sec (Figure 4C), as the ants changed from fairly direct and rapid walks toward the new nest to long, winding, and erratic paths throughout the arena (Figure 5).

### Experiment 3: Rotation of visual cues induces a corresponding rotation of headings

**Figure 6:**
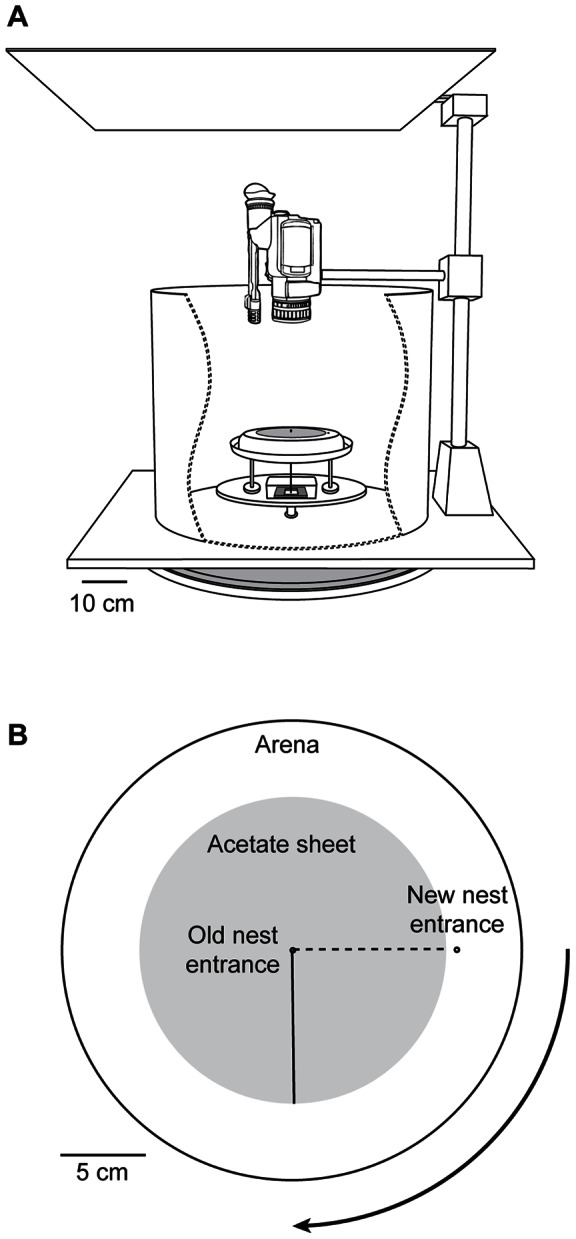
Apparatus for manipulation of visual cues during colony emigration. A) Side view shows the arena, old nest, and new nest arranged as in Figure 1, except that the arena was circular and designed to minimize visual cues (see Materials and Methods). The arena was centered in a cylinder with a complex visual scene displayed on its inner surface (partially cut away to show the arena). The cylinder rested on a copy stand that also carried a video camera and a screen to mask visual cues from the ceiling. The copy stand was mounted on a turntable so that it, along with the cylinder, camera, and screen, could be rotated around the stationary arena. B) Top view shows the arena floor. In the midst of each emigration, the copy stand was rotated 90° clockwise. Emigrating ants were observed to determine if they continued to walk toward the new nest (dashed line) or shifted their headings to match the new position of the visual scene (solid line). An acetate sheet was placed on the arena floor for consistency with the substrate-cue experiments, but it was not rotated.

The first two experiments suggested a paramount role for visual cues in orientation. To test this directly we modified the apparatus so that visual cues could be rotated around the arena (Figure 6). As before, we first waited until a focal ant had successfully recruited three times from the old to the new nest. We then rotated the visual surround 90° clockwise, while leaving the substrate in place.

**Figure 7:**
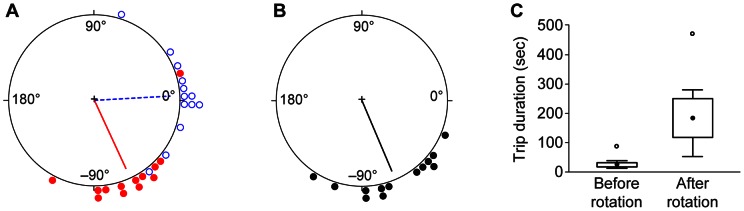
Rotation of visual cues strongly affects orientation by emigrating ants. A) The last trip before rotation (dashed blue line and open blue circles) was highly directed (Rayleigh test: r = 0.88, p = 6×10^−7^) and deviated only 3° from the direction of the new nest (0°). After rotation (solid red line and closed red circles), average heading remained highly directed (Rayleigh test: r = 0.88, p = 8×10^−7^) but shifted to 65° clockwise from the nest heading (Watson's two-sample test: U^2^  = 0.3883, p<0.001, n = 13). B) The average change in heading by each ant was 67° (r = 0.89, p = 5×10^−7^) and the mean change significantly differed from zero (Hotelling test: F_2, 11_  = 35.2, p = 2×10^−5^). C) The duration of journeys from the old to the new nest increased after rotation of visual cues (Wilcoxon paired ranks test: V = 2, n = 11, p = 0.0029). See Figure 2 for box plot details.

**Figure 8:**
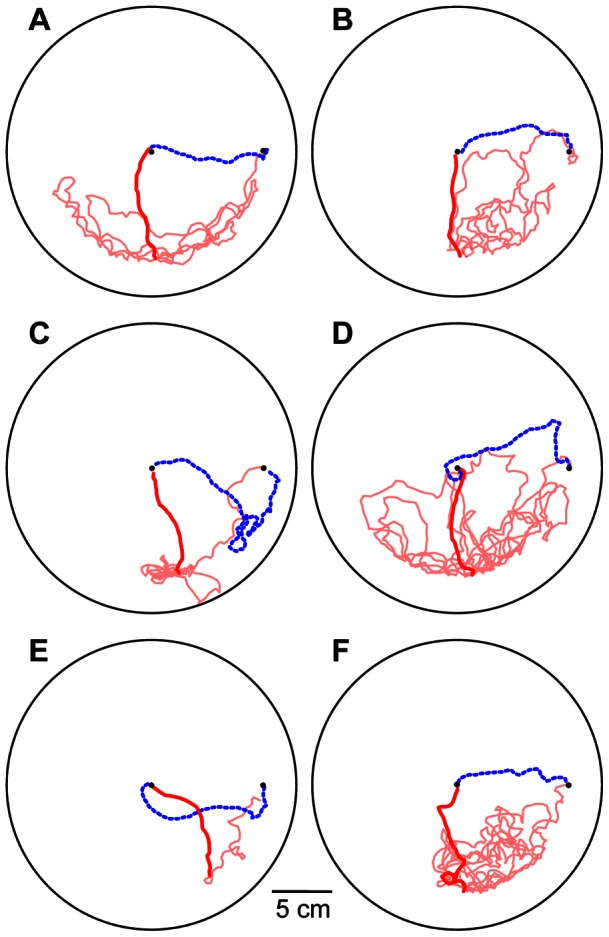
Sample paths before and after rotation of visual cues. Each panel shows a single ant's paths before rotation (dashed blue line) and afterwards (solid line). Paths after rotation are shown in red until they reach the edge of the acetate circle carpeting the arena, and in pink afterwards. Large circles show arena boundary. Small closed circles show locations of old nest entrance at arena center and new nest entrance at right side.

The result was a clear change in average heading in the same direction as the rotated visual cues (Figure 7A). Before rotation, headings were highly focused, with an average direction just 3° counterclockwise from the true bearing of the new nest. After rotation, the ants remained highly directed, but with an average heading 65° clockwise from the true bearing. The average change in heading across ants was 67° (Figure 7B), well above 0°, but somewhat less than the 90° rotation predicted if the ants' paths were determined entirely by the position of the rotated visual cues (95% confidence interval: 53° to 83°). The ants walked directly and rapidly from the arena center to the edge, reaching it in just 13±3 sec (Figure 8). Once there, not finding the expected nest, they began a winding search pattern that eventually brought them to the new nest entrance. These lengthy searches led to much longer trip durations after rotation (198±121 sec) than before (28±19 sec) (Figure 7C).

## Discussion

These results confirm and strengthen earlier work pointing to a dominant role for visual cues in navigation by *Temnothorax* ants [12], [16]–[18]. Indeed, we found no support for an influence of substrate-bound chemical cues on orientation. When the two sources of information were placed in competition, ants made a clear choice to rely on visual cues. Rotation of the substrate had no effect on headings as long as visual cues remained unchanged. Rotation of the visual surround, on the other hand, led to a corresponding rotation of headings, even though substrate cues remained in place. When visual cues were blocked, ants became highly disoriented, suggesting that they had no other way to orient.

It is possible that chemical cues would have been more important if we tested ants earlier in their learning of the route between old and new nests. Previous studies on other species suggest that naïve ants rely on pheromone information, but switch to visual cues as repeated trips familiarize them with the route [7]–[9], [11]. It is conceivable that *T. curvispinosus* follows a similar strategy. We tested transporters only after they had made several trips between the old and new nests, hence we cannot say whether they navigate differently in the earliest stages of route learning. Against this possibility, however, we found no sign of reliance on chemical cues, even when visual cues were removed. Ants simply became disoriented, taking long, circuitous search paths through the arena. This is in stark contrast to earlier studies of trail-laying species, where experienced foragers deprived of visual cues fell back on using the pheromone trail [10], [11], [13].

The lack of evidence for orientation by chemical marks may simply mean that ants did not deposit such marks in our experiments. This seems unlikely, given the evidence for substrate marking by several other *Temnothorax* species in similar contexts [12], [17]–[19], [30] and the extensive opportunity we gave transporters to mark their route before our manipulations. If the ants do in fact deposit chemicals, these may not be orientation trails that guide ants along specific routes. Instead, they may be a diffuse field of marks that identify an entire region as familiar territory. Similar behavior has been observed in *T. unifasciatus* [23] and in many other ants [24]–[26]. If transporters made such marks in our experiments, then rotating the field as we did in our first experiment would not be expected to cause a corresponding rotation of headings. An ant emerging into the arena center would still perceive a broad expanse of marked territory, rather than a narrow path guiding it in a particular direction. This would also explain the larger role for substrate cues found in earlier work on *T. unifasciatus* [16]. These ants were made to choose between marked and unmarked branches of a Y-bridge. Choosing the familiar-smelling branch automatically guides the ants on a particular heading, as a result of the substrate geometry. With our less-constrained arenas, diffusely applied marks would not provide such strong directionality. Earlier work on *T. albipennis* is also consistent with substrate markings serving as indicators of familiar territory rather than specific routes [17], [18]. Ants responded with hesitation and confusion when substrate markings were removed. They became reluctant to enter the now unmarked region, but eventually made their way across, likely guided by visual cues, and re-established well-directed traffic after about fifteen minutes.

Ants have many ways of using visual information for navigation, including path integration with a celestial compass, route-finding by local landmarks, and memorization of the entire visual surround [1]–[5]. In our experiments, ants had to rely on relatively distant cues, either the surrounding laboratory or a photographed panorama. The arenas themselves were largely devoid of landmarks that might pick out a specific route. Instead, as an ant walked from the old to the new nest, she may have learned the appearance of the distant panorama. On subsequent trips, she could choose a heading by moving so that the image projected on her retina replicated the memorized appearance of this panorama, a navigation strategy used by many other ant species [31]–[34]. These ants normally live on forest floors that offer both distant cues and nearby landmarks. Our experiments can say little about the role of nearby landmarks in navigation, but they do offer one hint of their importance. In the second experiment, when distant cues were obscured by a paper cylinder, the paths taken by the ants suggested their use of the arena itself as a landmark. That is, they tended to search the arena corners, perhaps because they visually resembled the corner location of the new nest entrance. Future work should more directly address the relative role of local landmarks and distant views.

Rotation of the visual panorama does not provide a full explanation of the ants' behavior in the third experiment. The average heading change was less than the 90° predicted by the size of the rotation. This may reflect subtle cues available in the arena itself, or lingering effects of the ants' orientation as they emerged into the arena center. Nonetheless, vision clearly had an overwhelming influence on the ants' headings, reinforcing earlier findings that even ants with small eyes and coarse visual acuity can be surprisingly reliant on visual cues for navigation.

## Materials and Methods

### Subjects

Colonies of *Temnothorax curvispinosus* were collected in Princeton, New Jersey on the grounds of the Institute for Advanced Study, with the Institute's permission. Colonies were found in hickory nut nests, and all colonies had at least one queen, 20–100 workers, and 30–50 brood items. Workers were marked with individually specific patterns of paint drops on the head, thorax, and gaster. Colonies were housed in cavity nests made from a balsa wood slat (2.4-mm thick) sandwiched between glass microscope slides (50×75 mm). A rectangular cavity (25×33 mm) was cut through the middle of the slat, and a 1.6 mm diameter entrance hole was drilled through the center of the roof. Colonies received water and an agar-based diet *ad libitum*.

Experiments 1 and 2 were conducted at Princeton University in the summer of 2005 using three colonies collected in July 2005. Experiment 3 was conducted at Arizona State University between October 2007 and April 2008, using three colonies collected in May 2007. Nineteen ants were tested in Experiment 1 (six to seven from each colony), nine in Experiment 2 (two to five from each colony), and twelve in Experiment 3 (three to six from each colony). Only one ant (in Experiment 3) was tested more than once; results were unchanged when this ant's second trial was excluded from the analysis. In all experiments, at least one day elapsed between trials on a given colony.

### Apparatus

For experiments 1 and 2 the arena was a 22.5 cm square plastic dish with 1.5-cm-high walls coated with Fluon to prevent the ants from escaping. The arena was mounted on risers directly above a small box containing the experimental colony in its old nest (Figure 1). From this box ants could climb a wooden dowel leading to a narrow brass tube (2 mm diameter) running through the center of the arena floor. The dowel was surrounded by a Fluon-coated, 27-mm diameter plastic cylinder. This barrier ensured that ants climbed through the brass tube to the arena floor, rather than across its bottom surface. In one corner of the arena, 13 cm from the central entrance, another hole led to a new nest attached to the bottom of the arena. The arena floor was carpeted with a clear acetate sheet, 20 cm in diameter. A hole in the center of the sheet allowed it to be threaded onto the central brass tube, around which it could be freely rotated. Faint markings were nicked into the edge of the sheet and the adjacent arena floor, to indicate a 60° rotation. A Sony DCR-TRV30 video camera mounted directly above the apparatus recorded all activity in the arena. Fluorescent ceiling fixtures were the only source of light.

Experiment 3 used a similar apparatus with modifications to allow rotation of the visual surround (Figure 6). The arena (with risers and nest box) stood in the center of a cardboard cylinder 1 m in diameter and 75 cm tall. The cylinder's inner surface was completely lined with a photograph of a complex visual scene (a panoramic view of the laboratory where the experiment was conducted; this scene was chosen to be comparable to the visual cues available to the ants in Experiments 1 and 2). The cylinder rested on a copy stand that also carried a Panasonic AG-HVX200 video camera and a translucent plastic screen (107×76 cm) that masked visual cues from the ceiling. The copy stand was mounted on a turntable so that it, along with the cylinder, camera, and screen, could be freely rotated around the arena. The arena itself remained stationary, because it was mounted on a pole that ran through holes in the copy stand and turntable to the bench below. For the first three replicates of this experiment, the arena consisted of a circular plastic dish (20 cm diameter) with 8-cm high Fluon-coated walls. This design avoided corners that might serve as local visual cues. To further minimize such cues, the remaining ten trials used an arena made from a plastic disk (27 cm diameter) rimmed with a Fluon-coated gutter that lay below the plane of the disk. The gutter prevented escapes, but its position kept it out of the ants' sight over most of the arena surface. For consistency with Experiments 1 and 2, the arena was carpeted with an acetate sheet (18 cm diameter), but this was not rotated during the experiment.

### Experimental procedure

Each trial was started by removal of the old nest's roof to induce emigration. Scouts soon entered the arena, discovered the new nest, and began recruiting nestmates with tandem runs and transports [20], [35], [36]. The identity of each recruiting ant was noted, and once an ant had completed three recruitments she was designated a focal ant for the trial. When this ant returned to the old nest to retrieve another recruit, the orientation cues were manipulated: for Experiment 1, the substrate was rotated 60° clockwise; for Experiment 2, the substrate was rotated 60° clockwise and a cylinder of white poster paper (∼60 cm tall and ∼30 cm diameter) was placed around the arena; for Experiment 3, the visual surround was rotated 90° clockwise. In Experiments 1 and 2 an observer stood near the apparatus at a fixed position that enabled him to see the arena. In Experiment 3 the observer stood next to the apparatus and looked over the cylinder edge to watch the ants. He shifted with the cylinder when it was rotated, in case the ants were using him as a visual cue. This precaution, as well as the apparatus design described above, ensured that all visual cues detectable from the arena were rotated. When the focal ant returned to the arena, her path was observed to see how it had changed in response to the manipulation. Once a colony had completed its emigration to the new nest, it was returned to its nest box. Colonies were tested more than once, but with an interval of several days between trials. A fresh new nest was built for each trial. Arenas and acetate sheets were re-used but were cleaned with ethanol or soap and water before each trial.

### Recording and measurement of headings

In each replicate, a video camera recorded all activity in the arena, while an observer verbally noted the identities of each ant. Clips of the focal ant's recruitment trips before and after manipulation were imported into Apple Motion, where each path was traced. To measure headings, the arena image was overlaid with a polar coordinate graph centered on the old nest entrance. Headings were measured relative to the true bearing of the new nest. In Experiments 1 and 3, the heading of each path was measured at 3, 6, and 9 cm from the center; in Experiment 2, headings were measured at 1.6, 3.2, 4.8, 6.4, 8.0, and 9.6 cm. Results were similar at all distances, and so are presented only for 9 cm (Experiments 1 and 3) or 6.4 cm (Experiment 2).

### Analysis

We compared the heading of the last recruitment trip before manipulation with that of the first recruitment trip after manipulation. For each set of paths, we calculated the average heading across ants as well as *r*, the length of the mean vector. This value measures directedness and can vary from 0 (headings are randomly dispersed) to 1 (all headings point in the same direction). In the figures, headings are shown on circular diagrams with a radial line pointing toward the average heading. The line's length corresponds to *r*, scaled so that a value of 1 extends fully to the circle.

Directedness of headings was assessed with a Rayleigh test. A Watson-Williams test was used to detect a difference between mean headings before and after manipulation. To control for variation among subjects, we also calculated for each ant the change between her heading before and after manipulation. A Hotelling test determined whether the mean change differed from zero. The duration of each trip was measured from the ant's emergence at the central hole to her entry into the new nest (except for two trials in Experiment 3 that could not be included because the ant left the camera's field of view before finding the new nest). All statistical analyses were performed in R [37].
