# Correction: Visual Navigation during Colony Emigration by the Ant *Temnothorax rugatulus*

**DOI:** 10.1371/journal.pone.0133455

**Published:** 2015-07-20

**Authors:** Sean R. Bowens, Daniel P. Glatt, Stephen C. Pratt

In the title and Abstract of this article, the wrong species name is given. The correct species name is *Temnothorax curvispinosus*. The correct title is: Visual Navigation during Colony Emigration by the Ant *Temnothorax curvispinosus*. The second sentence of the Abstract should read: “We evaluated the roles of both modalities during colony emigration by *Temnothorax curvispinosus*. The correct citation is: Bowens SR, Glatt DP, Pratt SC (2013) Visual Navigation during Colony Emigration by the Ant *Temnothorax curvispinosus*. PLoS ONE 8(5): e64367. doi:10.1371/journal.pone.0064367
